# The triglyceride-glucose index for diabetic retinopathy prediction in Chinese patients with type 2 diabetes: a preliminary cross-sectional study

**DOI:** 10.3389/fendo.2026.1784073

**Published:** 2026-03-19

**Authors:** Xiuping Qiu, Shidi Wen, Ruiyu Lin, Yang Chen, Jushun Zhang, Xiaolei Ni, Zhicheng Zhang

**Affiliations:** 1 Department of Endocrinology, Longyan First Affiliated Hospital of Fujian Medical University, Longyan, Fujian, China; 2 Department of Comprehensive Oncology Treatment, Longyan First Affiliated Hospital of Fujian Medical University, Longyan, Fujian, China; 3 Department of Orthopedics, Longyan Hospital of Tradictional Chinese Medical (TCM) Affiliated To Xiamen University, Longyan, China

**Keywords:** association analysis, cross-sectional study, diabetic retinopathy (DR), risk prediction, triglyceride-glucose (TyG) index, type 2 diabetes mellitus (T2DM)

## Abstract

**Objective:**

To evaluate the association between the TyG index and DR while critically assessing the predictive performance of this composite index. Specifically, we aimed to quantify the independent association between TyG and DR in different subgroups and determine the practical utility of TyG for clinical DR prediction.

**Methods:**

This cross-sectional study included 1,761 patients with T2DM visiting the National Metabolic Management Center. Biochemical parameters and results from fundus screening examinations were collected. Associations between the TyG index and DR were examined using multivariate logistic regression, smooth curve modeling, and stratified subgroup analyses. Receiver operating characteristic (ROC) curve analysis was conducted to evaluate the predictive performance of the TyG index.

**Results:**

The mean TyG index was significantly higher among patients with DR compared to those without DR. In multivariate regression, each one-unit increase in the TyG index was associated with a 1.57-fold higher likelihood of DR. Stratified analysis indicated that this association was more pronounced in female patients, younger patients, and those with a shorter disease duration. Smooth curve analysis demonstrated a nonlinear relationship, with a notable increase in DR risk when the TyG index exceeded 9.5. ROC curve analysis indicated an area under the curve of 0.5523 for the TyG index in predicting DR, with a specificity of 92.4% and a sensitivity of 28.2% at a cutoff value of 10.24.

**Conclusion:**

Although the TyG index is statistically associated with DR (OR = 1.57, p<0.0001), its extremely poor discriminatory ability (AUC = 0.5523, sensitivity=28.16%) renders it clinically unsuitable for DR screening, diagnosis. These findings suggest that predicting DR requires multi-parametric assessment incorporating metabolic, endocrine, inflammatory, and vascular markers, rather than reliance on a single composite index.

## 1 Introduction

Diabetes mellitus (DM) and its associated complications continue to pose significant global public health challenges (1, 2). Epidemiological data indicate a persistent upward trend in the prevalence of DM in China (3). In 2019, the global number of patients with DM was approximately 463 million, with projections estimating an increase to 700 million by 2045. In China, the number of patients with DM has reached 148 million, representing approximately 10.5% of the national population and ranking highest worldwide. Notably, a substantial increase in DM prevalence has been observed among patients aged 18–39 years (3). Diabetic retinopathy (DR), a serious chronic complication of DM, is strongly associated with DM duration. The global prevalence of DR is estimated at approximately 34.6%, with rates reaching up to 34.08% in certain regions, including areas of China (4). Among patients with a DM duration of 0–5 years, the prevalence of DR is approximately 6.6%; this figure rises to over 80% in those with a DM duration of ≥ 20 years (5, 6).

The triglyceride-glucose (TyG) index has emerged as a widely studied metabolic marker. The TyG index facilitates the rapid evaluation of insulin resistance by incorporating fasting blood glucose and triglyceride concentrations. In addition to reflecting baseline metabolic status, the TyG index has been associated with multiple chronic conditions (7). Existing evidence has demonstrated significant correlations between the TyG index and the incidence of cardiovascular disease, stroke, gestational DM, and other metabolic disorders (8, 9). A notable advantage of the TyG index is its capacity to identify underlying metabolic abnormalities and disease risk through the use of routinely available biochemical parameters.

Recent studies have provided new insights into the relationship between the TyG index and DR. Substantial evidence has demonstrated an association between elevated TyG index values and the presence of DR (10). A study involving 4,721 patients with diabetes conducted by Pan et al. identified the TyG index as an independent risk factor for DR (11). Furthermore, a large-scale meta-analysis comprising more than 19,000 participants systematically confirmed that higher TyG index levels are significantly associated with an increased risk of DR, highlighting its potential as a predictive marker for early detection of DR onset (12). Additional clinical studies, with sample sizes ranging from over 500 to more than 4,000 patients, have consistently validated this association. These studies indicate that the TyG index not only serves as an independent predictor for DR but is also correlated with the severity of retinal lesions (13–15). In addition, elevated TyG index values have been linked to an increased risk of other microvascular complications in patients with diabetes, including diabetic nephropathy and neuropathy. The index demonstrates synergistic interactions with conventional risk factors such as hypertension, hyperglycemia, and dyslipidemia, further amplifying disease risk (11). Collectively, these findings support the integration of the TyG index into early screening and risk stratification protocols for patients with diabetes, enabling earlier identification and more targeted management of complications such as DR.

In this study, an in-depth cross-sectional analysis was conducted to examine the association between the TyG index and DR in patients with DM managed at the National Metabolic Management Center (MMC) in Longyan City. The novelty of this study lies in its status as the first to systematically evaluate the predictive value of the TyG index for DR within a defined regional population, while applying rigorous control of potential confounding variables. The findings have the potential to contribute novel insights into the early risk assessment of DR and may provide a scientific basis for clinical prevention and personalized intervention strategies.

## 2 Methods

### 2.1 Study participants

This cross-sectional study was conducted at the National MMC of Longyan First Hospital between January 2022 and December 2023. A total of 1,761 patients with type 2 diabetes mellitus (T2DM) were included. The inclusion criteria were as follows: patients who (1) met the Chinese diagnostic criteria for T2DM, (2) were aged ≥ 18 years, and (3) voluntarily provided consent to participate in the study. The exclusion criteria (9) were defined as follows: patients diagnosed with (1) gestational diabetes, (2) diabetic ketoacidosis, (3) severe hepatic or renal dysfunction, (4) incomplete or missing key data related to exposure or outcome variables, (5) comorbidities involving other types of diabetes (e.g., type 1 diabetes, gestational diabetes, or specific forms of diabetes), (6) active infections, acute or chronic inflammation, or autoimmune diseases, (7) recent history of surgery or trauma, (8) prior ophthalmic procedures related to retinopathy such as laser photocoagulation or vitrectomy, (9) use of medications known to affect blood glucose or lipid levels (e.g., long-term glucocorticoids), or (10) retinopathy resulting from other causes, including hypertensive retinopathy or retinal vein occlusion. DM diagnosis was established in accordance with the Guideline for the Prevention and Treatment of T2DM in China (2020 edition), based on any of the following: fasting blood glucose ≥ 7.0 mmol/L, 2-hour post-load plasma glucose ≥ 11.1 mmol/L, or glycated hemoglobin (HbA1c) ≥ 6.5%. Data collection was carried out through the electronic medical record system and structured clinical assessments, with efforts made to ensure completeness and accuracy.

The outcome variable, DR, was diagnosed according to established criteria, including the International Clinical DR Disease Severity Scale and the guidelines issued by the American Academy of Ophthalmology (16). To ensure inter-rater reliability, all ophthalmologists involved in DR grading underwent standardized training based on the ETDRS protocol before the study. Additionally, a random sample of 10% of the retinal images was independently regraded by a second ophthalmologist, and the inter-rater agreement was assessed using Cohen’s kappa statistic, which showed excellent agreement (κ = 0.89).

Diagnosis was conducted based on the independent assessments of two or more qualified ophthalmologists using fundus imaging techniques, such as color fundus photography or fluorescein angiography. DR was classified as either non-proliferative diabetic retinopathy at various severity levels or proliferative diabetic retinopathy. Diagnostic classification was based on the identification of characteristic retinal lesions, including microaneurysms, retinal hemorrhages, hard exudates, neovascularization, and other established markers. That blood samples were collected via venipuncture in the morning after an overnight fast. Each participant provided a single blood sample at the time of enrollment.

### 2.2 Variables

The exposure variable was the TyG index, calculated as ln [fasting triglycerides (mg/dL) × fasting blood glucose (mg/dL)/2]. All measurements were performed within 24 hours of patient enrollment at the MMC, using standardized biochemical analyzers and calibrated reagent kits. In accordance with existing literature, the TyG index was analyzed both as a continuous variable and as a categorical variable, divided into tertiles, to comprehensively assess its association with DR (10). Covariates were selected based on prior research findings and established clinical physiological mechanisms. This included age, sex, disease duration, blood pressure, body mass index, visceral fat area, HbA1c, and lipid profile parameters.

### 2.3 Data processing

Missing data were addressed using multiple imputation to enhance the accuracy and completeness of statistical analyses. For continuous variables, mean imputation was applied, while for categorical variables, imputation was performed using the most frequently observed category. The proportion of missing data was below 5%, a threshold generally considered unlikely to introduce significant bias into the study outcomes. Most of the missing data were attributed to incomplete fundus examinations in some patients. To address missing covariate data, multiple imputations via chained equations were used, with 20 imputed datasets (m = 20). Participants with missing data related to exposure or outcome variables were excluded, as specified in the predefined study protocol. For continuous variables that were not normally distributed—including fasting and postprandial serum insulin and C-peptide levels—logarithmic transformation was applied using EmpowerStats software, with a formula of $Y=1n(\frac{X+\epsilon }{1-X+\epsilon })$. When variable values ranged between 0 and 1, the transformation occasionally produced negative values.

### 2.4 Ethical statement

Approval for this study was granted by the Ethics Committee of Longyan First Hospital (Approval No [2023]. Ethics Research No. 84), and all procedures were conducted in full compliance with the ethical principles for medical research outlined in the Declaration of Helsinki. Written informed consent was obtained from all participants prior to inclusion.

All data were anonymized to maintain confidentiality and were used solely for academic research purposes. The right to privacy of each patient was fully upheld throughout the study. Personal data were strictly protected, and data analysis was conducted only after complete de-identification.

### 2.5 Statistical methods

Continuous variables were expressed as mean ± standard deviation for data with a normal distribution or as median (minimum, maximum) for data with a skewed distribution. Categorical variables were presented as frequencies or percentages. For comparisons between groups, appropriate statistical tests were applied: Chi-squared tests were used for categorical variables, Student’s *t*-tests for normally distributed continuous variables, and Mann–Whitney U tests for non-normally distributed continuous variables.

All statistical analyses were performed using R statistical software. Two-tailed *p*-values < 0.05 were considered statistically significant.

A comprehensive investigation of the association between the TyG index and DR was carried out through a series of predefined analytical steps.

#### 2.5.1 Regression model analysis

Three hierarchical logistic regression models were constructed using a stepwise adjustment approach. Model 1 was specified as an unadjusted baseline model. Model 2 was adjusted for sociodemographic characteristics, while Model 3 was fully adjusted by incorporating all covariates listed in **Table 1**. This modeling strategy was employed to systematically assess the robustness of the association between the TyG index and DR under varying levels of covariate adjustment.

**Table 1:** Baseline characteristics of the study participants.

Variable	Non-DR (n=1413)	DR (n=348)	P-value
Age, years	54.93 ± 10.71	56.19 ± 10.07	0.047
Duration of diabetes, months	23.92 ± 53.91	30.64 ± 57.70	0.068
Diastolic blood pressure, mmHg	79.46 ± 10.59	79.28 ± 11.03	0.781
Systolic blood pressure, mmHg	129.05 ± 17.42	130.23 ± 17.61	0.259
Fasting Insulin,pmol/L	4.14 ± 1.24	4.26 ± 0.61	0.066
HbA1C, %	8.15 ± 2.14	8.14 ± 2.07	0.986
Postprandial insulin, pmol/L	5.88 ± 11.42	7.70 ± 40.18	0.138
TYG index	9.16 ± 0.74	9.46 ± 1.13	<0.001
Sex, n (%)			0.665
Female	935(66.2%)	226(64.9%)	
Male	478(33.8%)	122(35.1%)	
DR status, n (%)			<0.001
Without DR	1413 (100.0%)	0 (0.0%)	
With DR	0 (0.0%)	348(100.0%)	

DR, diabetic retinopathy; HbA1C, glycated hemoglobin; TYG, triglyceride-glucose index. Data are presented as mean ± SD or n (%).

#### 2.5.2 Nonlinear association assessment

To account for potential nonlinear relationships, generalized additive models (GAMs) were applied. Penalized splines were used to fit smooth curves, and recursive algorithms were implemented to identify inflection points, thereby allowing for the construction of a two-piecewise linear model.

Log-likelihood ratio comparisons between standard logistic regression models and piecewise linear models were performed to determine the model that best explained the association between the TyG index and DR.

#### 2.5.3 Subgroup and sensitivity analyses

Subgroup analyses were conducted using stratified logistic regression models and GAMs. Continuous stratification variables were converted into categorical variables based on clinical cutoff points or tertiles, and interaction terms were tested. Likelihood ratio tests were conducted to assess potential effect modification.

Sensitivity analyses were carried out. These included converting the TyG index into a categorical variable and calculating *p*-values for trend tests to confirm the consistency of findings derived from analyses using the TyG index as a continuous variable. These approaches were used to further examine potential nonlinear relationships.

## 3 Results

### 3.1 Characteristics of the study participants

A total of 1,761 patients with T2DM were included in the analysis, consisting of 600 males and 1,161 females. Among these patients, 348 were diagnosed with DR, while the remaining 1,413 were categorized as the non-DR group. The mean age of participants was 55.18 ± 10.60 years. The TyG index ranged from 7.05 to 12.94, with a median value of 9.16.

The mean age of patients in the DR group was found to be significantly higher than that of those in the non-DR group (56.19 ± 10.07 years vs. 54.93 ± 10.71 years; *p* = 0.047). The disease duration was slightly longer in the DR group compared to the non-DR group (30.64 ± 57.70 months vs. 23.92 ± 53.91 months), although the difference did not reach statistical significance (*p* = 0.068). No statistically significant differences were observed between the groups in diastolic blood pressure, systolic blood pressure, fasting insulin levels, postprandial insulin levels, or HbA1c levels, with all *p* > 0.05. However, the TyG index was significantly higher in the DR group than in the non-DR group (9.46 ± 1.13 vs. 9.16 ± 0.74; *p* < 0.001). Sex distribution did not differ significantly between the two groups (female: 66.2% vs. 64.9%; male: 33.8% vs. 35.1%; *p* = 0.665).

### 3.2 Univariate analysis of factors associated with DR

Univariate analysis demonstrated that each one-unit increase in the TyG index, analyzed as a continuous variable, was significantly associated with an increased risk of DR (odds ratio [OR] = 1.50; 95% confidence interval [CI]: 1.31–1.72; *p* < 0.0001). In tertile-based analysis, an elevated risk was observed in the highest TyG index tertile compared to the lowest; however, this difference did not reach statistical significance (OR = 1.26; *p* = 0.096). A statistically significant reduction in risk was observed in the intermediate tertile (OR = 0.65; *p* = 0.005). Sex did not have a significant effect on the risk of DR. A slight increase in risk was associated with each additional year of age (OR = 1.01; *p* = 0.047); however, this association was not statistically significant when age was analyzed as a categorical variable. The overall disease duration was not significantly associated with DR risk, though a significantly higher risk was observed among patients with the longest disease duration (OR = 1.50; *p* = 0.012). Elevated fasting and postprandial insulin levels were associated with an increased risk of DR, with the strongest association observed in the intermediate tertile group. Detailed results are presented in **Table 2**.

**Table 2:** Univariate analysis of factors associated with diabetic retinopathy.

Variable	Statistics (n, %)/Mean ± SD	OR (95% CI)	P-value
TYG	9.22 ± 0.84	1.50 (1.31, 1.72)	<0.0001
TYG tertile			
Low	587 (33.33%)	1.00 (reference)	—
Middle	582 (33.05%)	0.65 (0.48, 0.88)	0.0055
High	592 (33.62%)	1.26 (0.96, 1.66)	0.0961
Sex			
Female	1161 (65.93%)	1.00 (reference)	—
Male	600 (34.07%)	1.06 (0.83, 1.35)	0.6649
Age, years	55.18 ± 10.60	1.01 (1.00, 1.02)	0.0472
Age tertile			
Low	538 (30.55%)	1.00 (reference)	—
Middle	594 (33.73%)	1.04 (0.77, 1.40)	0.7953
High	629 (35.72%)	1.19 (0.89, 1.58)	0.2483
Duration of diabetes, mo	25.23 ± 54.72	1.00 (1.00, 1.00)	0.0700
Duration tertile			
Low	467 (33.36%)	1.00 (reference)	—
Middle	463 (33.07%)	0.95 (0.68, 1.34)	0.7736
High	470 (33.57%)	1.50 (1.09, 2.07)	0.0120
Diastolic BP, mmHg	79.42 ± 10.67	1.00 (0.99, 1.01)	0.7808
Diastolic BP tertile			
Low	578 (32.82%)	1.00 (reference)	—
Middle	529 (30.04%)	1.02 (0.75, 1.37)	0.9157
High	654 (37.14%)	1.12 (0.84, 1.48)	0.4397
Systolic BP, mmHg	129.28 ± 17.46	1.00 (1.00, 1.01)	0.2586
Systolic BP tertile			
Low	515 (29.24%)	1.00 (reference)	—
Middle	642 (36.46%)	0.93 (0.69, 1.25)	0.6431
High	604 (34.30%)	1.10 (0.82, 1.48)	0.5138
Fasting insulin	4.16 ± 1.14	1.08 (0.97, 1.21)	0.1479
Fasting insulin tertile			
Low	585 (33.22%)	1.00 (reference)	—
Middle	577 (32.77%)	1.52 (1.13, 2.04)	0.0054
High	599 (34.01%)	1.36 (1.01, 1.83)	0.0402
HbA1C, %	8.14 ± 2.12	1.00 (0.95, 1.06)	0.9863
HbA1C tertile			
Low	543 (30.83%)	1.00 (reference)	—
Middle	611 (34.70%)	1.17 (0.88, 1.57)	0.2865
High	607 (34.47%)	1.06 (0.79, 1.42)	0.7174
Postprandial insulin	6.24 ± 20.58	1.00 (1.00, 1.01)	0.2132
Postprandial insulin tertile			
Low	578 (32.82%)	1.00 (reference)	—
Middle	595 (33.79%)	1.79 (1.34, 2.41)	<0.0001
High	588 (33.39%)	1.35 (1.00, 1.84)	0.0516

OR, odds ratio; CI, confidence interval; SD, standard deviation; BP, blood pressure; HbA1C, glycated hemoglobin; TYG, triglyceride-glucose index.

### 3.3 Stratified analysis of the association between the TyG index and DR in different subgroups

The stratified analysis further clarified that the association between the TyG index and the risk of DR differed across various patient subgroups (**Table 3**). The specific findings are presented as follows:

**Table 3:** Stratified analysis of the association between TYG and diabetic retinopathy (DR) in different subgroups.

Subgroup	N	OR (95% CI)	P-value
Sex			
Female	1161	1.88 (1.59, 2.21)	<0.0001
Male	600	0.85 (0.65, 1.12)	0.2466
Age tertile			
Low	538	2.07 (1.63, 2.64)	<0.0001
Middle	594	2.04 (1.57, 2.66)	<0.0001
High	629	0.87 (0.68, 1.12)	0.2933
Disease duration tertile (months)			
Low	467	2.04 (1.53, 2.71)	<0.0001
Middle	463	1.28 (0.98, 1.67)	0.0728
High	470	1.23 (0.95, 1.59)	0.1167
Diastolic BP tertile			
Low	578	1.55 (1.21, 1.99)	0.0005
Middle	529	1.20 (0.92, 1.56)	0.1884
High	654	1.68 (1.36, 2.08)	<0.0001
Systolic BP tertile			
Low	515	1.74 (1.36, 2.23)	<0.0001
Middle	642	1.45 (1.15, 1.83)	0.0018
High	604	1.36 (1.08, 1.71)	0.0080
Fasting insulin tertile			
Low	585	1.93 (1.48, 2.51)	<0.0001
Middle	577	1.41 (1.15, 1.73)	0.0010
High	599	1.23 (0.96, 1.59)	0.1050
HbA1c tertile			
Low	543	1.41 (1.07, 1.85)	0.0144
Middle	611	1.23 (0.96, 1.58)	0.1015
High	607	1.96 (1.56, 2.46)	<0.0001
Postprandial insulin tertile			
Low	578	2.33 (1.78, 3.04)	<0.0001
Middle	595	1.37 (1.11, 1.71)	0.0038
High	588	1.19 (0.92, 1.53)	0.1769

In sex-stratified analysis, a significant positive association between the TyG index and DR was observed among female patients (OR = 1.88; 95% CI: 1.59–2.21; *p* < 0.0001), while no statistically significant association was identified among male patients (OR = 0.85; 95% CI: 0.65–1.12; *p* = 0.2466). When stratified by age, significant associations were found in both the younger (OR = 2.07; 95% CI: 1.63–2.64; *p* < 0.0001) and middle-aged groups (OR = 2.04; 95% CI: 1.57–2.66; *p* < 0.0001), whereas no significant association was observed in the older age group (OR = 0.87; *p* = 0.2933).

In the stratification by disease duration, a strong association between the TyG index and DR was found in patients with the shortest disease duration (OR = 2.04; *p* < 0.0001). The strength of the association decreased in the intermediate-duration group (OR = 1.28; *p* = 0.0728) and the long-duration group (OR = 1.23; *p* = 0.1167), with neither reaching statistical significance. When stratified by diastolic blood pressure, significantly elevated risks of DR were observed among patients in both the lowest (OR = 1.55; *p* = 0.0005) and highest (OR = 1.68; *p* < 0.0001) blood pressure groups. Subgroup analysis by systolic blood pressure indicated statistically significant positive associations between the TyG index and DR across all tertiles (low, intermediate, and high), with *p* < 0.05 in each group.

The results of the stratified analyses by fasting serum insulin and HbA1c levels indicated that the TyG index exerted particularly significant effects on DR in subgroups with low fasting serum insulin levels (OR = 1.93; *p* < 0.0001) or elevated HbA1c levels (OR = 1.96; *p* < 0.0001). Further cross-stratification analysis, based on tertiles of the TyG index, demonstrated that among female patients, those in the highest TyG index tertile exhibited a significantly increased risk of DR (OR = 1.66; 95% CI: 1.18–2.34; *p* = 0.0038). In contrast, no significant association was observed in male patients (*p* > 0.05). Stratified analysis by age indicated that a high TyG index was a strong risk factor for DR among patients in both the young and middle-aged tertile groups (young group: high tertile OR = 1.99; *p* = 0.0090; middle-aged group: high tertile OR = 2.04; *p* = 0.0050). However, in older patients, the association was reversed, with a high TyG index linked to a significantly reduced risk of DR (OR = 0.52; *p* = 0.0107). Additionally, potential interaction effects or, in some cases, protective effects between the TyG index and DR were observed within several subgroups stratified by disease duration, blood pressure, fasting or postprandial insulin levels, and HbA1c levels. These effects, however, were not uniform and varied considerably across subgroups.

In summary, the predictive value of the TyG index for DR was primarily evident among female patients, younger age groups, patients with a shorter disease duration, those with extreme blood pressure levels, and patients presenting with elevated fasting serum insulin or HbA1c levels.

### 3.4 Multivariate regression analysis of factors associated with DR

Multivariate regression analysis demonstrated a significant positive association between the TyG index and the risk of DR, as presented in **Table 4**. In the unadjusted model, each one-unit increase in the TyG index was associated with a 1.50-fold higher risk of DR (OR = 1.50; 95% CI: 1.31–1.72; *p* < 0.0001). This association remained robust after adjustment for potential confounders, including sex, age, disease duration, blood pressure, fasting and postprandial serum insulin levels, and HbA1c levels (Adjust I: OR = 1.55; 95% CI: 1.32–1.83; *p* < 0.0001). Following further smoothing of these covariates (Adjust II), the association between the TyG index and DR risk remained statistically significant (OR = 1.57; 95% CI: 1.32–1.87; *p* < 0.0001). These findings indicated that an elevated TyG index may serve as an independent risk factor for DR and may carry clinical relevance in risk assessment.

**Table 4:** Association between TYG and diabetic retinopathy (DR): Multivariable regression analyses.

Exposure	Non-adjusted	Adjust I	Adjust II
TYG (continuous)	1.50 (1.31, 1.72) <0.0001	1.55 (1.32, 1.83) <0.0001	1.57 (1.32, 1.87) <0.0001
TYG Tertile			
Low (reference)	1.00	1.00	1.00
Middle	0.65 (0.48, 0.88) 0.0055	0.63 (0.44, 0.89) 0.0089	0.56 (0.39, 0.81) 0.0019
High	1.26 (0.96, 1.66) 0.0961	1.29 (0.93, 1.78) 0.1266	1.24 (0.88, 1.75) 0.2258

In the subgroup analyses stratified by TyG index tertiles, the results indicated that, compared to patients in the lowest tertile group, those in the intermediate tertile group exhibited the lowest risk of DR, which was statistically significant (Adjust II: OR = 0.56; 95% CI: 0.39–0.81; *p* = 0.0019). Although an OR > 1 was observed in the highest tertile group (Adjust II: OR = 1.24; 95% CI: 0.88–1.75; *p* = 0.2258), the association did not reach statistical significance. These findings indicated that moderate levels of the TyG index may be associated with a reduced risk of DR, whereas excessively elevated levels could be linked to an increased risk, although not statistically confirmed in this model.

### 3.5 Smooth curve analysis of the association between the TyG index and the risk of DR

**Figure 1:**
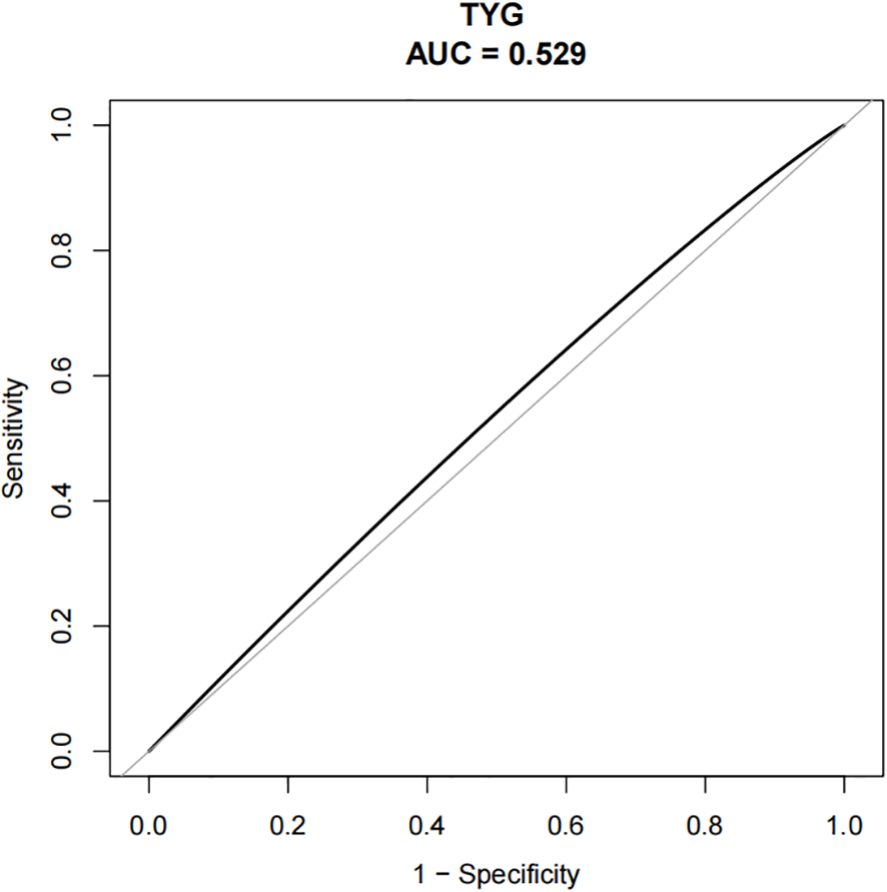
Nonlinear association between the TyG index and the risk of DR in patients with diabetes.

Smooth curve analysis indicated a nonlinear relationship between the TyG index and the risk of DR, as presented in **Figure 1**. Within the lower to moderate range of TyG index values (approximately 7.0 to 9.5), the risk of DR remained relatively low and stable. However, when the TyG index exceeded approximately 9.5, a marked increase in DR risk was observed, with a steep upward trend. The lowest point on the fitted curve was identified at a TyG index value near 9.5, suggesting this level may correspond to the minimal observed risk exposure. In the higher TyG index range (> 9.5), the 95% confidence intervals widened considerably on both sides of the curve, indicating stronger associations but increased estimation uncertainty at elevated TyG index levels. Overall, these findings indicated the presence of a potential threshold effect, with a TyG index of approximately 9.5 representing a critical inflection point. Beyond this value, the risk of DR appeared to increase substantially, underscoring the need for further investigation into whether patients with TyG index levels above this threshold may benefit from more intensive DR monitoring.

### 3.6 Interaction analysis between the TyG index and the risk of DR

Interaction analysis demonstrated significant differences in the association between the TyG index and the risk of DR across various subgroups (**Table 5**). The effect of the TyG index on DR risk was more pronounced among female patients (OR = 1.88; 95% CI: 1.59–2.21; *p* < 0.0001), younger patients (OR = 2.07; 95% CI: 1.63–2.64), those with a shorter disease duration, lower postprandial serum insulin levels, and extreme HbA1c levels. In these subgroups, the interaction *p*-values were all < 0.05, indicating statistically significant effect modification. In contrast, no significant interaction effects were observed when the analysis was stratified by diastolic or systolic blood pressure.

**Table 5:** Interaction analysis between TYG and diabetic retinopathy (DR) across subgroups.

Modifier	Min P (terms)	P for interaction
Age tertile	<0.0001	<0.0001 ***
Sex	<0.0001	<0.0001 ***
Postprandial insulin tertile	<0.0001	0.0006 ***
Duration (month) tertile	<0.0001	0.0186 *
HbA1C% tertile	<0.0001	0.0195 *
Fasting insulin tertile	<0.0001	0.0479 *
Diastolic BP tertile	<0.0001	0.1350
Systolic BP tertile	<0.0001	0.3421

*Statistically significant difference.

***Highly statistically significant difference.

### 3.7 ROC curve analysis of the TyG index for predicting the risk of DR

**Figure 2:**
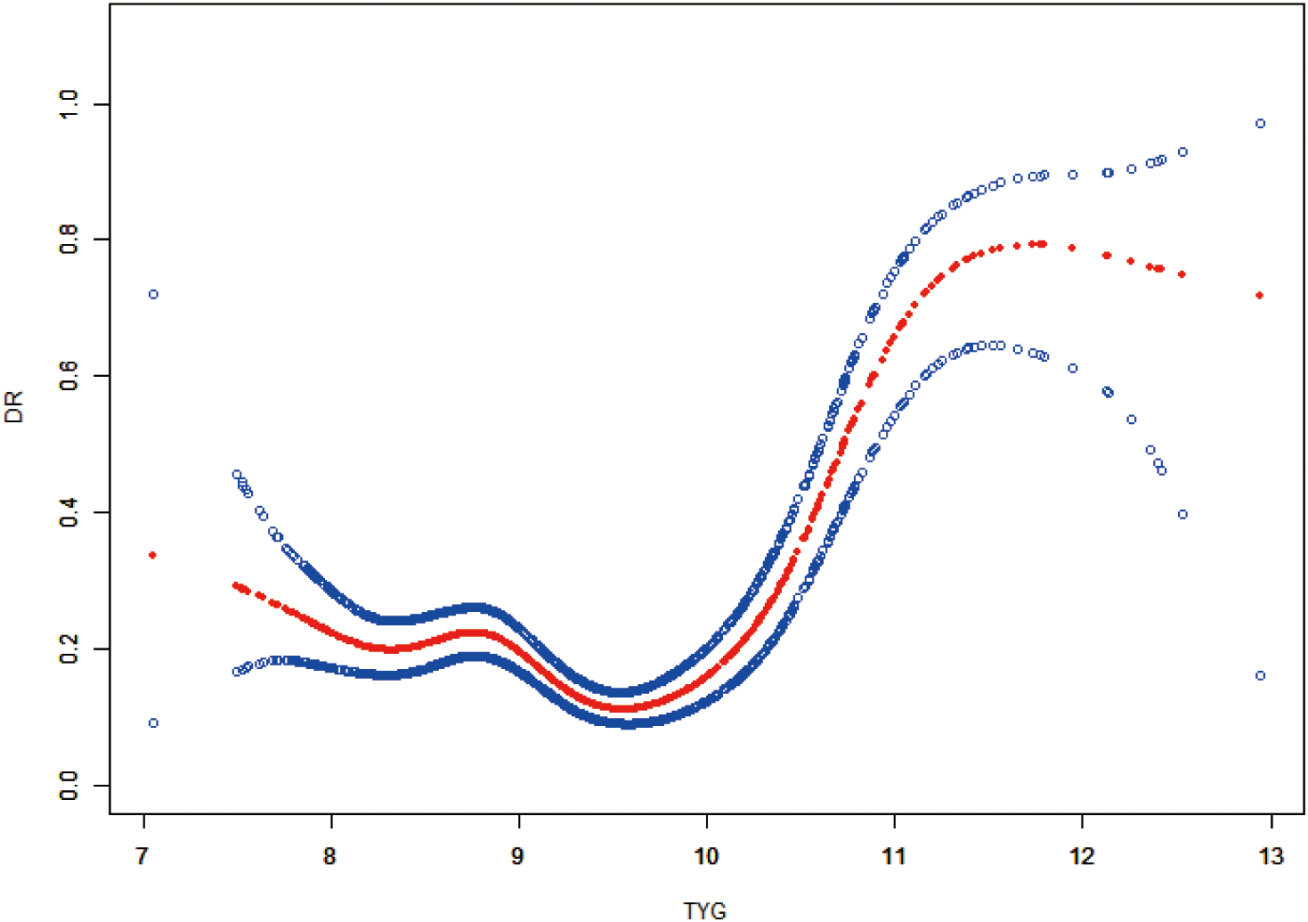
ROC Curve of the TyG Index for Predicting DR.

ROC curve analysis was conducted to assess the diagnostic performance of the TyG index in predicting DR (**Figure 2**). The area under the curve (AUC) for the TyG index was calculated as 0.5523 (95% CI: 0.5143–0.5903), indicating limited discriminatory ability. At an optimal cutoff value of 10.2433, specificity was 92.43%, while sensitivity was 28.16%. At this threshold, diagnostic accuracy was 79.73%, the positive predictive value was 47.80%, and the negative predictive value was 83.93%. The positive likelihood ratio was 3.72, the negative likelihood ratio was 0.78, and the diagnostic OR was 4.78.

These findings indicated that, although the TyG index demonstrated high specificity suggesting potential use in excluding patients without DR, it exhibited limited sensitivity for discovering patients with DR. As such, the TyG index presented limited effectiveness as a stand-alone screening tool. The statistical significance of the AUC further supported the conclusion that the TyG index had a restricted ability to discriminate between patients with and without DR.

Therefore, the TyG index may be more appropriate as a supplementary component within comprehensive risk assessment models, rather than as an independent diagnostic indicator. In clinical applications, its use may be enhanced when combined with other established risk factors to support integrated DR risk assessment.

## 4 Discussion

There are many risk factors for diabetes, including obesity, insulin resistance, Dietary Patterns, increased cortisol, and Chronic Psychological Stress. Obesity is a major driver of insulin resistance. Excessive adipose tissue, particularly visceral fat accumulation, increases circulating free fatty acids and inflammatory cytokines (TNF-α, IL-6), which directly impair insulin signaling pathways. High-calorie, high-refined carbohydrate, and high-saturated fat diets promote insulin resistance. Excessive intake of sugary beverages and processed foods contributes to dyslipidemia and impaired glucose tolerance. Chronic stress also impairs glucose homeostasis and increases abdominal fat deposition. This factors may contribute to rising TyG levels.

In this study, the association between the TyG index and DR was systematically examined. By using a large sample of consecutively enrolled patients with T2DM, both representativeness and statistical power were enhanced. A significant positive association was observed between elevated TyG index values and an increased risk of DR. Findings from the smooth curve analysis and GAMs indicated a nonlinear relationship, in which DR risk progressively increased with rising TyG index values. Notably, the risk elevation became significantly steeper when the TyG index exceeded approximately 9.5, indicating a potential threshold effect. Regarding diagnostic performance, the area under the ROC curve for the TyG index was 0.5523 (95% CI: 0.5143–0.5903). At an optimal cutoff value of 10.2433, specificity reached 92.43%, while sensitivity was limited to 28.16%, indicating restricted discriminatory capacity when the TyG index was applied as a stand-alone diagnostic marker. However, the high specificity suggested potential utility in identifying patients at low risk of DR. These results were generally consistent with findings from previous domestic and international studies. Stratified analyses revealed contradictory associations-for example, protective effects for older patients and patients with longer disease duration. We acknowledge that these results should be interpreted with caution and do not necessarily indicate true protection, but may reflect complex interactions of disease duration, age-related physiological changes, and cumulative treatment exposure in the study cohort.

We have cited prior studies that identified similar thresholds associated with increased cardiometabolic risk, insulin resistance, and endothelial dysfunction. For example, studies by Tao LC et al. and Akbar MR et al. have reported TyG index values above 8.5-9.2 as indicative of significant insulin resistance and higher risk for type 2 diabetes and cardiovascular events. SO that a threshold near 9.5 may represent a more advanced state of metabolic dysregulation where the cumulative impact on vascular health becomes significantly pronounced, potentially explaining the observed nonlinear increase in brachial-ankle pulse wave velocity (baPWV) (7, 17).

For example, in a study involving 799 Chinese patients with T2DM, the TyG index was independently associated with DR after adjustment for confounding variables such as age, sex, disease duration, and blood glucose levels (9). Furthermore, each one-unit increase in the TyG index was significantly correlated with elevated DR risk, and higher TyG quartiles were positively associated with increased DR prevalence. Another investigation, which included 1,141 patients with T2DM, reported that the TyG index served as a useful marker for DR risk stratification, particularly among individuals with suboptimal glycemic control, thereby supporting its potential role in identifying patients who were at high-risk.

The TyG index, as a surrogate marker of insulin resistance, has been considered a more effective indicator of disturbances in glucose-lipid metabolism, an underlying metabolic abnormality closely implicated in the pathogenesis and progression of DR (7, 9). An elevated TyG index reflects more severe insulin resistance, which has been linked to the progression of DR through multiple pathological mechanisms. Insulin resistance has been strongly associated with endothelial dysfunction, chronic low-grade inflammation, and increased oxidative stress. These pathophysiological changes contribute to damage to the retinal microvasculature, including thickening of the capillary basement membrane, increased microvascular permeability, and impaired retinal microcirculation (18–23). Additionally, a higher TyG index is often accompanied by hypertriglyceridemia and mild hyperglycemia. These metabolic disturbances may act synergistically to promote endothelial injury within retinal microvessels, thereby exacerbating inflammatory responses and pathological neovascularization, ultimately facilitating the progression of DR.

Notably, the TyG index, being influenced by insulin sensitivity, captures metabolic imbalances that may not be adequately identified through fasting blood glucose or lipid levels alone. It has been regarded as more sensitive than conventional metabolic parameters in identifying early risk associated with DR. In summary, by reflecting key pathophysiological processes such as insulin resistance, inflammatory activation, and dyslipidemia, the TyG index provides a mechanistic basis for assessing DR risk. These findings offer theoretical support for the early identification of at-risk patients and for the development of targeted intervention strategies aimed at preventing diabetic complications.

This study may hold practical clinical relevance for the early identification and management of DR risk. By focusing on the TyG index, a simple, cost-effective, and readily accessible metabolic marker, novel perspectives on individualized and precise risk stratification were introduced for patients with DM. Unlike prior studies that primarily emphasized conventional glycemic or lipid parameters, the present investigation, supported by a large sample size, rigorous stratification, and interaction analyses, offered initial, comprehensive, and multidimensional insights into the clinical use of the TyG index across diverse population subgroups. Notably, the TyG index demonstrated advantages in the high-specificity exclusion of individuals without DR risk.

These findings may support early identification of high-risk individuals in primary care settings and in resource-constrained regions, inform optimized referral and follow-up strategies, and contribute to the development of integrated chronic disease management frameworks. Although the TyG index cannot replace existing imaging-based screening modalities, it may offer considerable potential as an adjunctive tool for preliminary screening and longitudinal follow-up in chronic disease care, particularly in rural health facilities where fundus imaging equipment is not readily available.

Several methodological strengths were present in the study design and data analysis strategy employed in the current investigation. First, the study was based on a large-scale, consecutively enrolled cohort of patients with DM, which enhanced both the representativeness of the findings and the statistical power. All primary variables were collected and measured using standardized protocols, with comprehensive quality control procedures implemented to ensure data accuracy and consistency. Second, stratified subgroup analyses and interaction analyses were incorporated to comprehensively assess the effects of the TyG index on the risk of DR and to examine potential heterogeneity across subgroups. These approaches may have contributed to greater credibility and robustness of the results. In addition, multiple statistical methods were applied to adjust for confounding factors, thereby minimizing potential sources of bias as effectively as possible. Finally, the applicability of the TyG index was systematically assessed as a low-cost and accessible biomarker across various clinical contexts, potentially providing novel reference points for risk stratification in the management of chronic diseases.

Several limitations were present in this study. First, patients with severe hepatic or renal dysfunction, acute complications, pregnancy, or other serious systemic conditions were excluded based on the inclusion and exclusion criteria; as a result, the findings may not be generalizable to these specific populations. Additionally, the single-center design, in which all participants were enrolled from a single hospital, may have limited the generalizability and external validity of the results. Outcomes may differ in studies conducted within other healthcare systems or geographic regions. Furthermore, the study population primarily consisted of Chinese adults; therefore, caution is warranted when extrapolating these findings to other ethnic groups or age categories.

Given the observational nature of the study, only an association between the TyG index and DR was established, without evidence of a causal relationship. These limitations highlight the need for future multicenter, large-scale, prospective studies involving diverse populations to further validate and extend the present findings.

## 5 Conclusion

Although the TyG index is statistically associated with DR (OR = 1.57, p<0.0001), its extremely poor discriminatory ability (AUC = 0.5523, sensitivity=28.16%) renders it clinically unsuitable for DR screening, diagnosis. These findings suggest that predicting DR requires multi-parametric assessment incorporating metabolic, endocrine, inflammatory, and vascular markers, rather than reliance on a single composite index. The potential clinical use of the TyG index in predicting chronic complications of DM is expected to expand substantially with continued exploration of the underlying mechanisms and the implementation of large-scale, multicenter cohort studies.

## Data Availability

The raw data supporting the conclusions of this article will be made available by the authors, without undue reservation.

## Glossary

T2DM: Type 2 Diabetes Mellitus
DR: Diabetic Retinopathy
TyG: Triglyceride-Glucose Index
ROC: Receiver Operating Characteristic
AUC: Area Under the Curve
OR: Odds Ratio
CI: Confidence Interval
MMC: Metabolic Management Center
BMI: Body Mass Index
FPG: Fasting Plasma Glucose
TG: Triglyceride
HDL-C: High-Density Lipoprotein Cholesterol
LDL-C: Low-Density Lipoprotein Cholesterol
HbA1c: Glycated Hemoglobin
SBP: Systolic Blood Pressure
DBP: Diastolic Blood Pressure
SD: Standard Deviation
HbA1C: glycated hemoglobin
GAM: Generalized Additive Model
